# Organization and Variation Analysis of 5S rDNA in Different Ploidy-level Hybrids of Red Crucian Carp × Topmouth Culter

**DOI:** 10.1371/journal.pone.0038976

**Published:** 2012-06-18

**Authors:** Weiguo He, Qinbo Qin, Shaojun Liu, Tangluo Li, Jing Wang, Jun Xiao, Lihua Xie, Chun Zhang, Yun Liu

**Affiliations:** Key Laboratory of Protein Chemistry and Fish Developmental Biology of Education Ministry of China, College of Life Sciences, Hunan Normal University, Changsha, People’s Republic of China; Virginia Tech Virginia, United States of America

## Abstract

Through distant crossing, diploid, triploid and tetraploid hybrids of red crucian carp (*Carassius auratus* red var., RCC♀, Cyprininae, 2n = 100) × topmouth culter (*Erythroculter ilishaeformis* Bleeker, TC♂, Cultrinae, 2n = 48) were successfully produced. Diploid hybrids possessed 74 chromosomes with one set from RCC and one set from TC; triploid hybrids harbored 124 chromosomes with two sets from RCC and one set from TC; tetraploid hybrids had 148 chromosomes with two sets from RCC and two sets from TC. The 5S rDNA of the three different ploidy-level hybrids and their parents were sequenced and analyzed. There were three monomeric 5S rDNA classes (designated class I: 203 bp; class II: 340 bp; and class III: 477 bp) in RCC and two monomeric 5S rDNA classes (designated class IV: 188 bp, and class V: 286 bp) in TC. In the hybrid offspring, diploid hybrids inherited three 5S rDNA classes from their female parent (RCC) and only class IV from their male parent (TC). Triploid hybrids inherited class II and class III from their female parent (RCC) and class IV from their male parent (TC). Tetraploid hybrids gained class II and class III from their female parent (RCC), and generated a new 5S rDNA sequence (designated class I–N). The specific paternal 5S rDNA sequence of class V was not found in the hybrid offspring. Sequence analysis of 5S rDNA revealed the influence of hybridization and polyploidization on the organization and variation of 5S rDNA in fish. This is the first report on the coexistence in vertebrates of viable diploid, triploid and tetraploid hybrids produced by crossing parents with different chromosome numbers, and these new hybrids are novel specimens for studying the genomic variation in the first generation of interspecific hybrids, which has significance for evolution and fish genetics.

## Introduction

Polyploidization, the addition of an extra set (or sets) of chromosomes to the genome, is a predominant mechanism for speciation in plants and animals [1]–[2]. Polyploidization can occur via somatic doubling, the fusion of unreduced gametes, and by means of a triploid bridge or polyspermy [1]–[3]. The current prevailing opinion is that hybridization plays an important role in triggering polyploidization [1], [4]–[6]. Polyploid species are particularly frequent in the plant kingdom [2], [7], and 40–70% of all plant species are polyploids [3], [8]. However, while it is generally relatively rare in animals [9]–[10], polyploidy has occurred extensively, independently, and is often repeated in many groups of fish, from the sharks to the higher teleosts [11]–[13]. Furthermore, artificially induced polyploidy has been used in aquaculture to produce sterility and to improve production [14].

Polyploidy in fish represents a useful model system with which to test theories about the origin and consequences of polyploidy that have been derived from work on plants [15]. Using RCC (*Carassius auratus* red var.) and TC (*Erythroculter ilishaeformis* Bleeker), three new types of different ploidy-level hybrid fish were successfully obtained. This is the first report on the formation of these viable diploid, triploid and tetraploid hybrids by crossing different parents with a different chromosome number in vertebrates.

**Table 1:** Examination of chromosome number in RCC, TC, 2nRT, 3nRT and 4nRT hybrids.

Fish type	Distribution of chromosome number									
	<48^a^	48	<100^a^	100	<74^a^	74	<124^a^	124	<148^a^	148
TC	12	188								
RCC			7	193						
2nRT					32	168				
3nRT							45	155		
4nRT									14	186

a The chromosome number is less than what they should be, owning to the loss of chromosomes in the procedure of chromosome preparation.

In eukaryotes, the 5S rDNA multigene family occurs as several thousands copies of tandem repeated units, comprising a highly conserved coding sequence of 120 bp and a flanking region of variable nontranscribed spacer (NTS) containing some regulatory elements for the transcription of the coding sequence [16]–[18]. Studies of the structural and functional organization of the 5S rRNA genes have been carried out in fungi [19]–[22], plants [23]–[24], animals [25]–[26], and particularly fish [17]–[18], [27]–[37]. Accumulation of molecular data from fish shows the occurrence of two 5S rDNA arrays characterized by a distinct NTS [18], [28], [30], [33]. This seems to imply a general trend of possessing two 5S rDNA classes for the organization of the 5S rRNA gene in the fish genome [38]. Moreover, in some bony fishes, oocyte- and somatic-type 5S rRNA genes are expressed differently in oocytes and somatic cells [26], [39]. Nevertheless, there are also reports of one or three 5S rDNA arrays in bony fishes [40].

**Figure 1:**
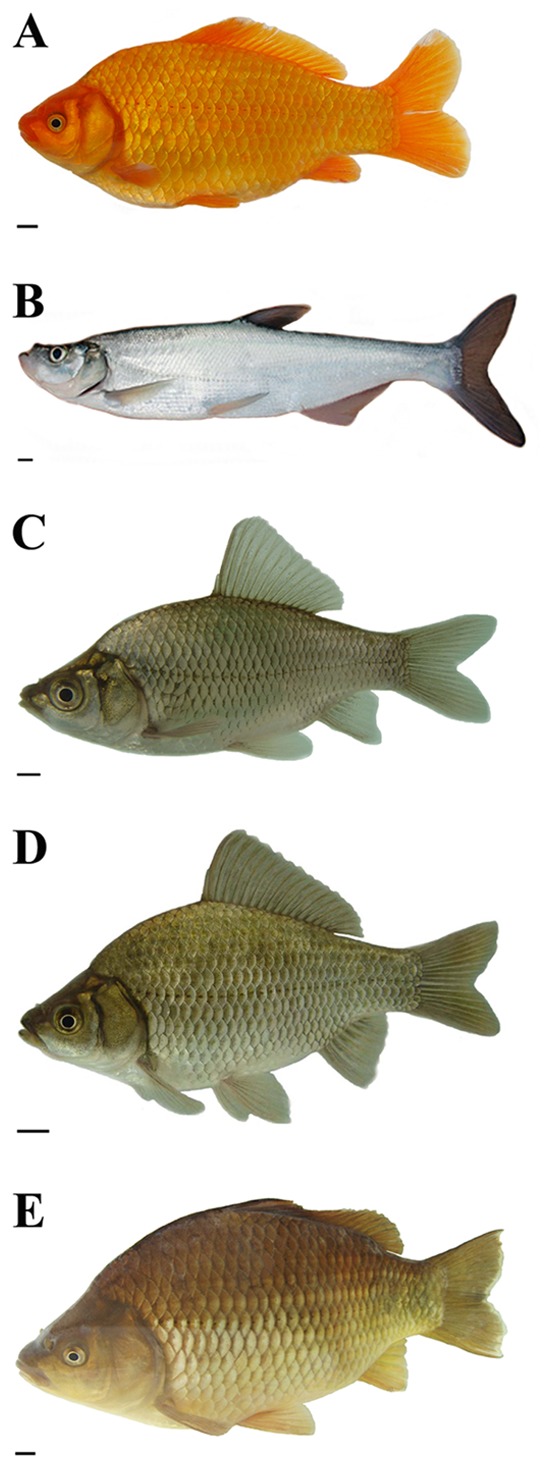
Appearance of RCC, TC and their hybrid offspring. (A) RCC. (B) TC. (C) 2nRT hybrids of RCC♀×TC♂. (D) 3nRT hybrids of RCC♀×TC♂. (E) 4nRT hybrids of RCC♀×TC♂. Scale bar in A–E, 1 cm.

**Figure 2:**
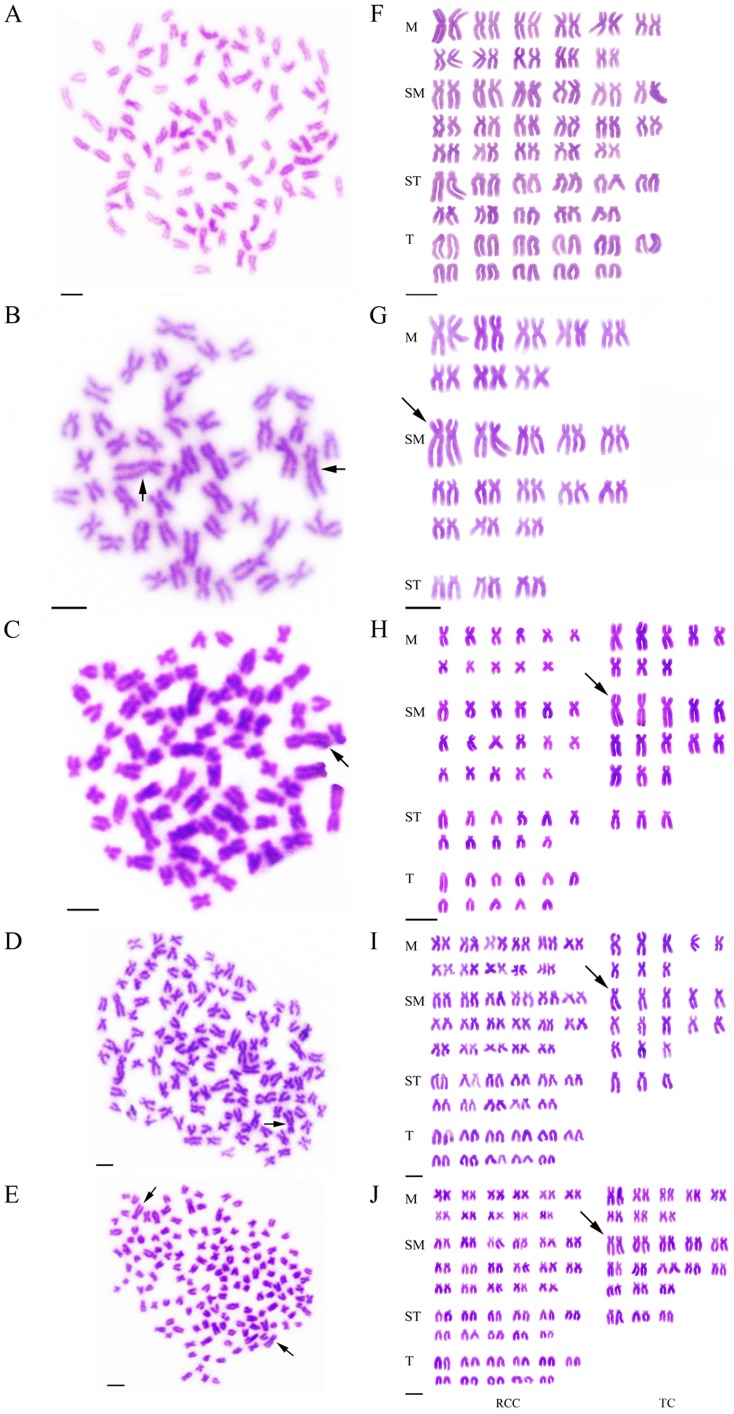
Chromosome spreads at metaphase and corresponding karyotypes of RCC, TC, and their hybrid offspring. (A) The 100 chromosomes of RCC, with no large submetacentric chromosome. (B) The 48 chromosomes of TC, with a pair of the largest submetacentric chromosomes indicated (solid arrows). (C) The 74 chromosomes of 2nRT hybrids, with a piece of the largest submetacentric chromosome indicated (solid arrow). (D) The 124 chromosomes of 3nRT hybrids, with a piece of the largest submetacentric chromosome indicated (solid arrow). (E) The 148 chromosomes of 4nRT hybrids, with a pair of the largest submetacentric chromosomes indicated (solid arrows). (F) The karyotype of RCC, in which no large submetacentric chromosome is detected. (G) The karyotype of TC, which includes a pair of the largest submetacentric chromosomes (solid arrow). (H) The karyotype of 2nRT hybrids, comprising one set of chromosomes from RCC and one set from TC. The solid arrow indicates a piece of the largest submetacentric chromosome, which is similar to that of TC. (I) The karyotype of 3nRT hybrids, consisting of two sets of chromosomes from RCC and one set from TC. The solid arrow indicates a piece of the largest submetacentric chromosome, which is similar to that of TC. (J) The karyotype of 4nRT hybrids, consisting of two sets of chromosomes from RCC and two sets from TC. The solid arrow indicates a pair of the largest submetacentric chromosomes similar to those of TC. Scale bar in A–J, 3 µm.

To further reveal the influence of hybridization and polyploidization on the 5S rDNA organization, we performed a study of nucleotide sequences and molecular organization of 5S rDNA in red crucian carp (RCC), topmouth culter (TC) and their hybrid offspring.

**Table 2:** Mean DNA content of RCC, TC, 2nRT, 3nRT and 4nRT hybrids.

		Ratio	
Fishtype	Mean DNAcontent^a^	Observed	Expected
RCC	101.29		
TC	67.40		
2nRT	84.19	2nRT/(0.5 RCC+0.5 TC) = 0.99^b^	1
3nRT	136.17	3nRT/(RCC+0.5 TC) = 1.01^b^	1
4nRT	163.01	4nRT/(RCC+TC) = 0.97^b^	1

a The intensity of fluorescence (unit, channel).

b The observed ratio was not significantly different (*P*>0.05) from the expected ratio.

**Figure 3:**
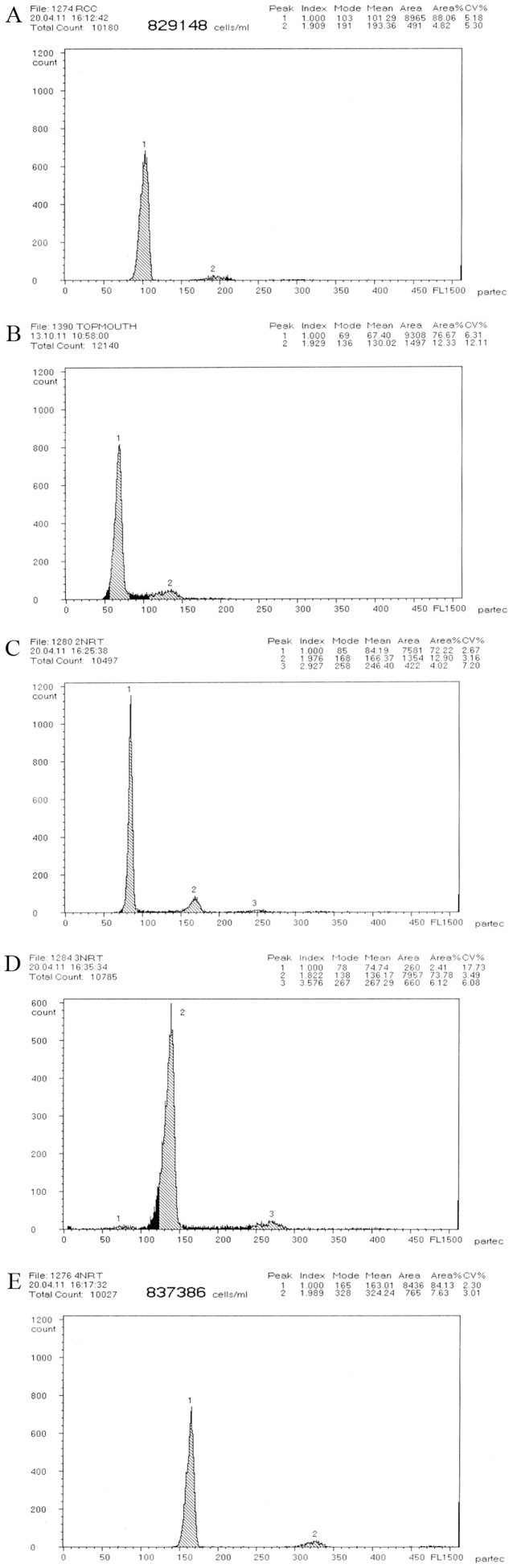
Cytometric histograms of RCC, TC and their hybrid offspring. (A) The mean DNA content of RCC (peak 1∶101.29). (B) The mean DNA content of TC (peak 1∶67.40). (C) The mean DNA content of 2nRT hybrids (peak 1∶84.19). (D) The mean DNA content of 3nRT hybrids (peak 2∶136.17). (E) The mean DNA content of 4nRT hybrids (peak 1∶163.01).

## Materials and Methods

### Ethics Statement

Administration of Affairs Concerning Animal Experimentation guidelines state that approval from the Science and Technology Bureau of China and the department of wildlife administration is not necessary when the fish in question are not rare or near extinction (first-class or second-class state protection level). Therefore, approval was not required for the experiments conducted in this paper.

**Figure 4:**
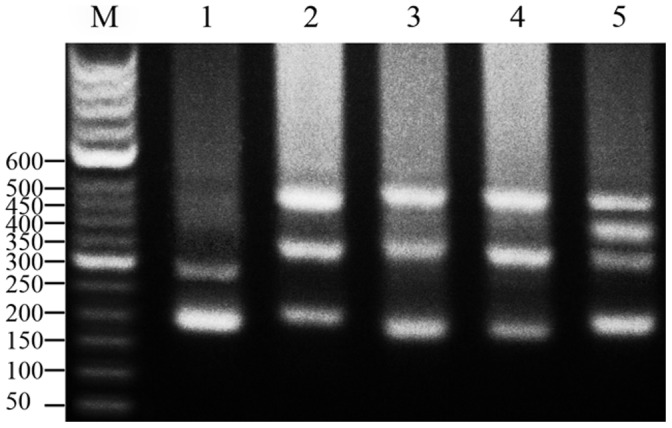
DNA bands amplified from RCC, TC and their hybrid offspring. M: DNA ladder markers (50 bp increments); lane 1: two DNA bands (∼200 and 300 bp) from TC; lane 2: three DNA bands (∼200, 350 and 500 bp) from RCC; lane 3: three DNA bands (∼200, 350 and 500 bp) from 2nRT hybrids; lane 4: three DNA bands (∼200, 350 and 500 bp) from 3nRT hybrids; lane 5: four DNA bands (∼200, 350, 400 and 500 bp) from 4nRT hybrids.

### Animals and Crossing Procedure

Specimens of RCC and TC were obtained from the Engineering Research Center of Polyploid Fish Breeding and Reproduction of State Education Ministry at Hunan Normal University. During the reproductive seasons (from June to July) in 2009, 2010 and 2011, 10 mature females and 10 mature males of both RCC and TC were chosen as the maternal and paternal parents, respectively. The crossings were performed by two groups. In the first group, RCC was used as the maternal, and TC as the paternal, While in the second group this was reversed. Mature eggs were fertilized and the embryos developed in the culture dishes at the water temperature of 20°C–22°C. In each crossing, 2000 embryos were chosen at random for the examination of the fertilization rate (number of embryos at the stage of gastrula/number of eggs×100%), the hatching rate (number of hatched fry/number of eggs×100%) and the adulthood rate (number of adulthood/number of eggs×100%). By using the same methods, the same-species mating of RCC and TC were used as controls. The hatched fry were then transferred to a special pond for further culture.

**Table 3:** The results of sequences.

samples	Number ofsequencedclones	PCR bands				
		∼200 bp	∼300 bp	∼350 bp	∼400 bp	∼500 bp
RCC	30	Ten clones of 203 bp	Absent	Ten clones of 340 bp	Absent	Ten clones of 477 bp
TC	20	Ten clones of 188 bp	Ten clones of 286 bp	Absent	Absent	Absent
2nRT	60	12 clones of 179 bp;8 clones of 203 bp	Absent	20 clones of 340 bp	Absent	20 clones of 495 bp
3nRT	60	20 clones of 188 bp	Absent	20 clones of 340 bp	Absent	20 clones of 495 bp
4nRT	80	20 clones of 203 bp	Absent	20 clones of 340 bp	20 clones of 406 bp	20 clones of 477 bp

The cross between RCC♀×TC♂ resulted in three ploidy-level hybrid offspring: diploid, triploid and tetraploid hybrids. In the reverse cross between TC♀×RCC♂ no living progeny were produced. Hereinafter, the diploid hybrids of RCC♀×TC♂ are abbreviated as 2nRT hybrids, the triploid hybrids of RCC♀×TC♂ as 3nRT hybrids, and the tetraploid hybrids of RCC♀×TC♂ as 4nRT hybrids.

**Figure 5:**
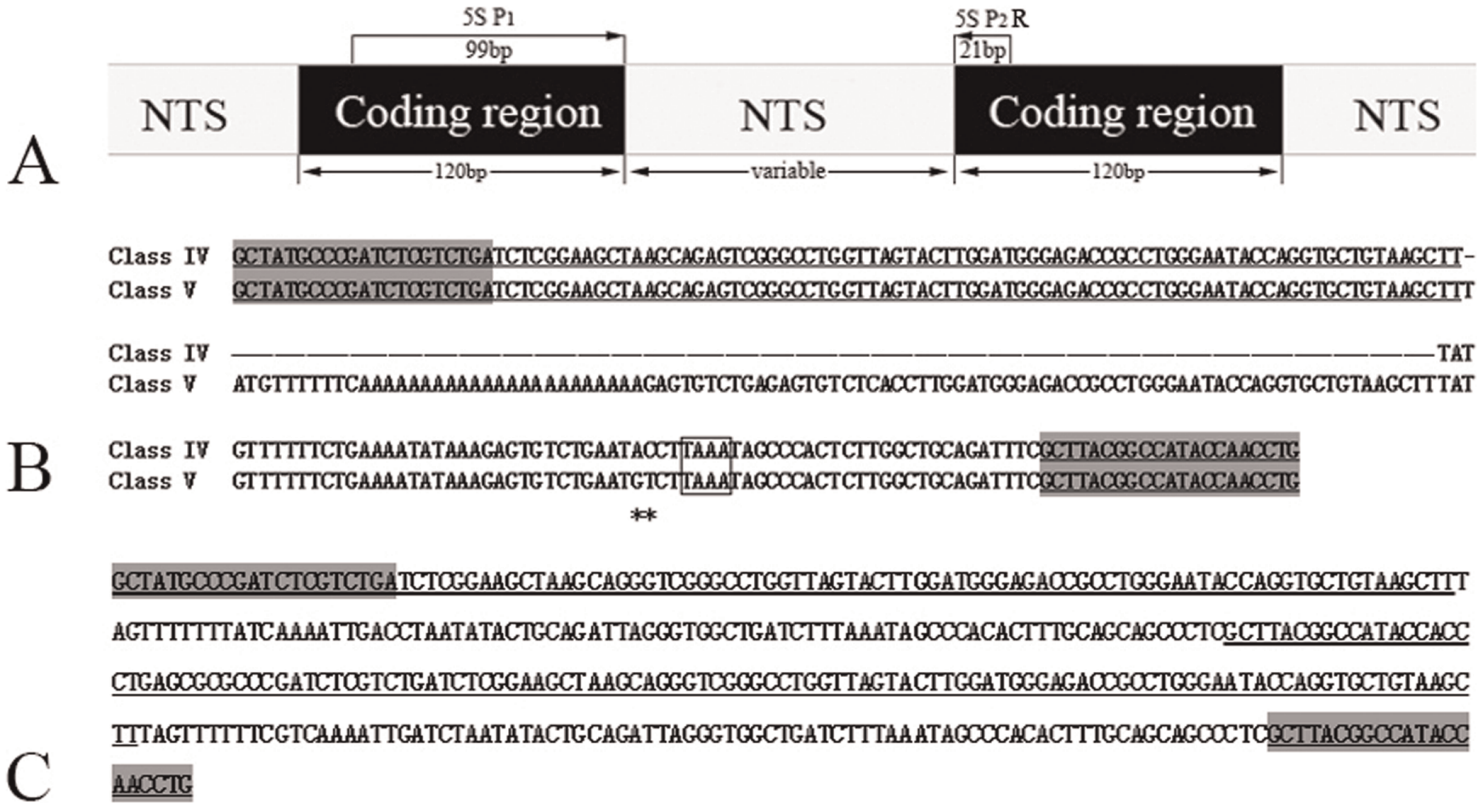
Representative sequences of monomeric 5S rDNA and dimeric 5S rDNA. (A) Arrangement of eukaryotic 5S rDNA and the illustration of the PCR amplification with 5S primers P_1_ and P_2_R; (B) Representative sequences of 5S rDNA Class IV and Class V from TC; (C) The dimeric 5S rDNA tandem arrays (NTS–I–N) of 4nRT hybrids. The gene sequences of 5S rDNA are underlined and the shaded regions show the 5S primers. Dashes indicate alignment gaps; asterisks represent variable sites; TATA element (TAAA) is included in box.

### Preparation of Chromosome Spreads and Measurement of DNA Content

To determine ploidy, chromosome counts were carried out on kidney tissue from 10 RCC, 10 TC, 10 2nRT, 10 3nRT and 10 4nRT hybrids at 1 year of age. Preparations were made according to the method of Liu et.al (2007) [12] with minor modifications. After culture for 2–3 days at the water temperature of 20°C–22°C, the samples were injected with concanavalin one to three times at a dose of 6–10 µg/g body weight. The interval time of injection was 12–24 hours. 2–3 hours prior to dissecting, each sample was injected with colchicines at a dose of 6–8 µg/g body weight. The kidney tissue was ground in 0.9% NaCl, followed by hypotonic treatment with 0.075 M KCl at 37°C for 40–60 min and then fixed in 3∶1 methanol-acetic acid for three changes. Cells were dropped on cold, wet slides and stained with 4% Giemsa for 30–45 min. The shape and number of chromosome were analyzed under a light microscope. 200 metaphase spreads of chromosomes were analyzed for each type of fish (20 from each sample). Preparations were examined under an oil lens at a magnification of ×330 and good-quality metaphase spreads were photographed for analysis of karyotypes. Lengths of entire chromosomes (long and short arms) were measured. Chromosomes were classified on the basis of their long-arm to short-arm ratios according to the reported standards [41].

**Figure 6:**
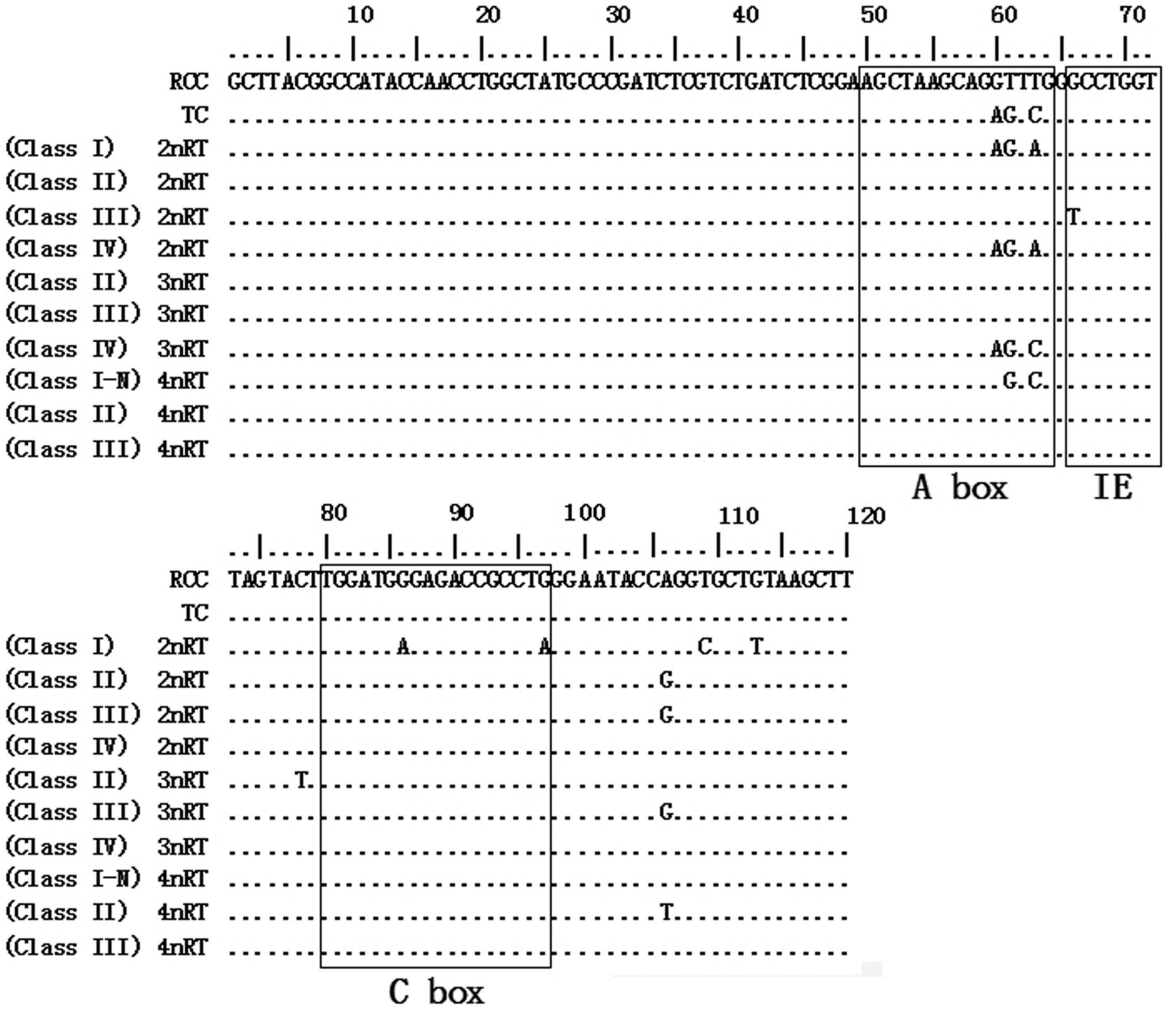
Complete 5S rRNA genes of RCC, TC and their hybrid offspring. Dots indicate the identical nucleotides and ICRs are included in the boxes.

The DNA content of erythrocytes of RCC, TC and their hybrid offspring were measured using a flow cytometer (Cell Counter Analyzer, Partec, Germany). 1–2 ml of blood was gathered from the caudal vein using a syringe containing ∼200–300 units of sodium heparin. Blood samples were then treated according to the method outlined in Liu et.al (2007) [12], and measured under the same condition. The DNA content of RCC and TC were used as the controls. To calculate the ratios of the DNA content of the hybrid offspring to the sum of that of RCC and TC, a χ2 test was used, with Yate’s correction applied to test for deviation from expected ratio values.

**Figure 7:**
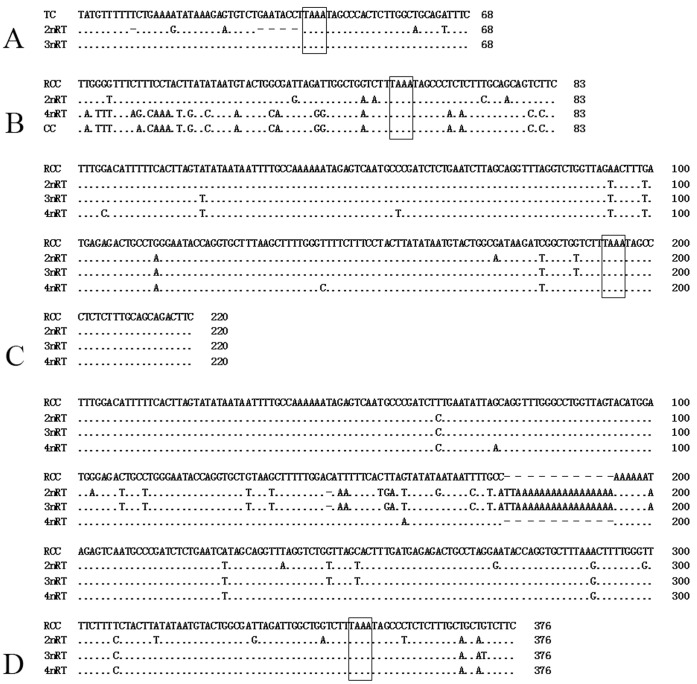
Alignment results of the NTS sequences from CC, RCC, TC and their hybrid offspring. (A) The 59 bp and 68 bp NTS sequences (NTS–IV) of TC, 2nRT and 3nRT hybrids; (B) NTS–I from RCC, CC, 2nRT and 4nRT hybrids; (C) NTS–II from RCC, 2nRT, 3nRT and 4nRT hybrids; (D) NTS–III from RCC, 2nRT, 3nRT and 4nRT hybrids. The NTS upstream TATA-like sequences are included in boxes. Dots indicate sequence identity and hyphens represent insertions/deletions.

### Genomic DNA Extraction, PCR and Sequencing

Total genomic DNA of RCC, TC and their hybrid offspring were extracted from the peripheral blood cells using a phenol/chloroform extraction method as described in Sambrook et al. (1989) [42]. A set of primers based on those described by Qin et al. [40] (5S P1, 5′-GCTATGCCCGATCTCGTCTGA-3′: 5S P2R, 5′- CAGGTTGGTATGGCCGTAAGC-3′) were designed and synthesized to amplify the 5S rRNA genes and their nontranscribed spacer regions directly from genomic DNA. PCR reactions were carried out in a volume of 25 µL with approximately 20 ng of genomic DNA, 1.5 mM of MgCl_2_, 250 µM of each dNTP, 0.4 µM of each primer, and 1.25 U of Taq polymerase (TaKaRa, Dalian, China). The thermal program consisted of an initial denaturation step at 94°C for 5 min, followed by 25 cycles (94°C for 30 sec, 60°C for 30 sec, and 72°C for 1 min) and a final extension step at 72°C for 10 min. Amplification products were separated on a 1% agarose gel using TBE buffer. The DNA fragments were purified using a gel extraction kit (Sangon Biotech Co., Ltd., Shanghai, China) and ligated into the pMD18-T vector (TaKaRa, Dalian, China). The plasmids were transformed into *E. coli* DH5a and purified, and the inserted DNA fragments were sequenced using an automated DNA sequencer (ABI PRISM 3730, Applied Biosystems, Carlsbad, CA). To determine sequence homology and variation among the fragments amplified from RCC, TC, 2nRT, 3nRT and 4nRT hybrids, sequences were aligned using BioEdit [43] and Clustal W [44].

**Figure 8:**
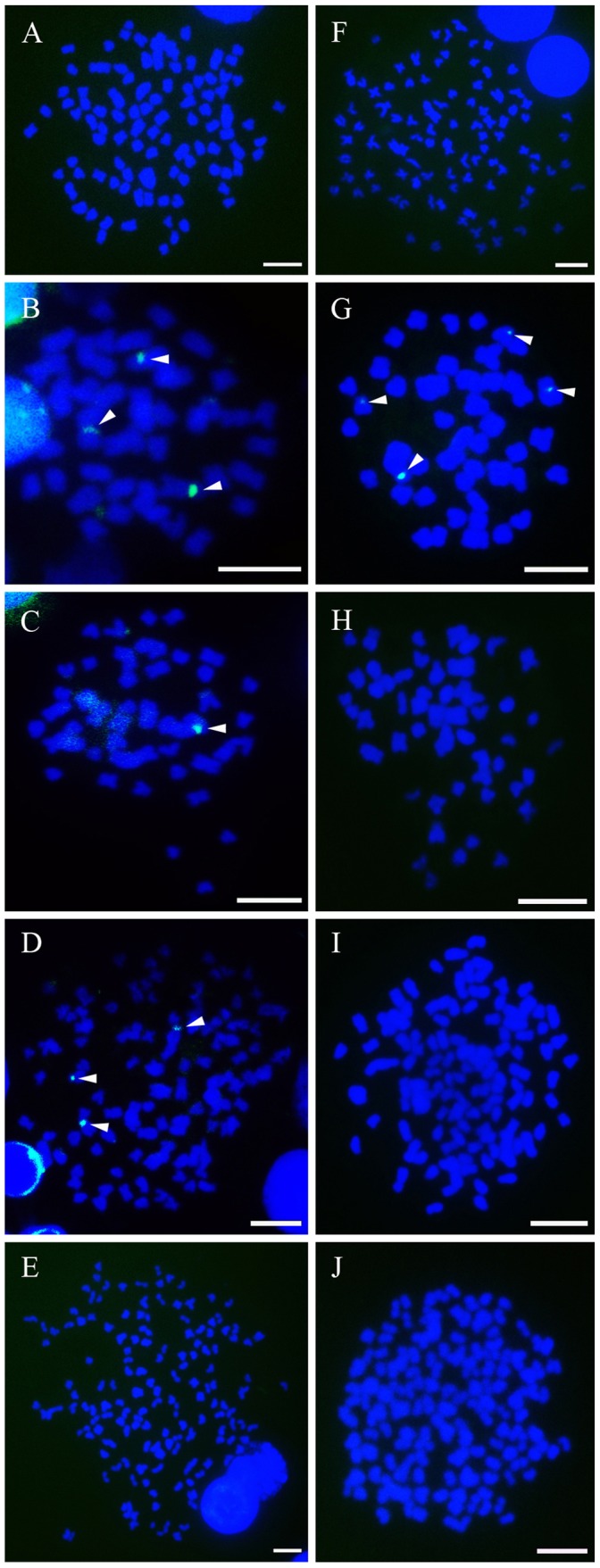
Fluorescence photomicrographs of mitotic metaphase chromosomes of RCC, TC and their hybrid offspring. Signals were detected with fluorescein isothiocyanate (FITC)-conjugated avidin and all the metaphase chromosomes were stained with DAPI. A–E (mitotic metaphase chromosomes of RCC, TC, 2nRT, 3nRT and 4nRT hybrids respectively) show the single-label FISH results hybridized with a probe from the cloned repeated fragments of class IV; F–J (mitotic metaphase chromosomes of RCC, TC, 2nRT, 3nRT and 4nRT hybrids respectively) demonstrate the hybridization results after single-label FISH with a probe from the cloned repeated fragments of class V. The white arrowheads indicate the fluorescent signals (green) of 5S rDNA. Scale bar in A–J, 3 µm.

### Fluorescence *in situ* Hybridization and Microscopy

Purified PCR products of 5S rDNA labeled with Dig-11-dUTP (Roche, Germany) were used as probes, and hybridization was performed according to the method described by Yi et al. (2003) [45] with minor modifications. After treatment with 30 µg/ml RNase A in 2×SSC for 30 min at 37°C, the slides with chromosome metaphase spreads were denatured in 70% deionized formamide/2×SSC for 2 min at 70°C, dehydrated in a 70%, 90% and 100% ethanol series for 5 min each (1×SSC is 0.15 M NaCl/0.015 M sodium citrate, pH 7.6), and then air-dried. 4 µl of the hybridization mixture (approximately 100 ng of labeled probes, 50% formamide, 10 mg dextran sulfate/ml and 2×SSC) was denatured for 10 min in boiling water, applied to the air-dried slides carrying denatured metaphase chromosomes under a 22×22 mm coverslip, and sealed with rubber cement. The slides were then put in a moist chamber and allowed to incubate overnight at 37°C.

**Table 4:** Nucleotide homology of NTS sequences among CC, RCC, TC, 2nRT, 3nRT and 4nRT hybrids (percentage).

NTS types (bp)	TC and 2nRT	TC and 3nRT	RCC and 2nRT	RCC and 3nRT	RCC and 4nRT	CC and 4nRT
59, 68	80.8	100	Absent in both	Absent in both	Absent in both	Absent in both
83	Absent in TC	Absent in both	92.7	Absent in 3nRT	73.8	97.5
220	Absent in TC	Absent in TC	97.2	97.2	96.3	Absent in CC
357, 375	Absent in TC	Absent in TC	86.4	88.8	97.7	Absent in 4nRT

Following overnight incubation, the coverslips were removed and the slides were rinsed at 43°C in: 2×SSC with 50% formamide, twice, 15 min each; 2×SSC, 5 min; 1×SSC, 5 min, then air-dried. The spectrum signals were achieved by application of 8 µl of 5 µg/ml FITC-conjugated antidigoxigenin antibody from sheep (Roche, Germany) and a final incubation in the humidity chamber at 37°C. After a series of washes with TNT (containing 0.1 M Tris-HCl, 0.15 M NaCl, 0.05% Tween 20) at 43°C, the slides were mounted in antifade solution containing 2 µg/ml 4',6-diamidino-2-phenylindole (DAPI) for 5 min. Slides were viewed under a Leica inverted CW4000 microscope and a Leica LCS SP2 confocal image system (Leica, Germany). Captured images were colored and overlapped in Adobe Photoshop cs4. At least 30 metaphases from each sample were analyzed.

## Results

### Fertilization Rate, Hatching Rate and Adulthood Rate

In the cross of RCC♀×TC♂ we observed a high fertilization rate (83.6%) and hatching rate (74.6%) but relatively low adulthood rate (28.6%). While in the same-species mating, the fertilization rate, hatching rate and adulthood rate of RCC were 89.9%, 85.4%, 75.5%, respectively, and that of TC were 94.9%, 89.8%, 77.4%. There were no living progeny in the reverse crosses between TC♀×RCC♂. Approximately 3000 2nRT, 2500 3nRT and 20 4nRT hybrids were obtained each year.

At the age of 2 years, because of the few number of 4nRT hybrids, only an artificial propagation of 2nRT and 3nRT hybrids was conducted during the reproductive season of last year, and neither 2nRT nor 3nRT hybrids were able to mate. According to our previous studies [11]–[12], we supposed that 4nRT hybrids were fertile though the confirmation of fertility or sterility requires further study.

### Chromosome Number and Karyotypes

The chromosome numbers were determined for the mitotic metaphases of all samples. Table 1 shows the distribution of chromosome number in RCC, TC, 2nRT, 3nRT and 4nRT hybrids. For diploid RCC (Figure 1A), 96.5% of chromosomal metaphases had 100 chromosomes with the karyotype formula of 22 m +34 sm +22 st +22 t (Figure 2A, F). For diploid TC (Figure 1B), 94% of chromosomal metaphases possessed 48 chromosomes with the karyotype formula of 16 m +26 sm +6 st (Figure 2B, G). In the hybrid offspring of RCC♀×TC♂ without barbels and with low body height (Figure 1C), 84% of chromosomal metaphases had 74 chromosomes with the karyotype formula of 19 m +30 sm +14 st +11 t (Figure 2C, H). In the hybrid offspring of RCC♀×TC♂ without barbels but having high body height (Figure 1D), 77.5% of chromosomal metaphases had 124 chromosomes with the karyotype formula of 30 m +47 sm +25 st +22 t (Figure 2D, I). In the hybrid offspring of RCC♀×TC♂ with barbels (Figure 1E), 93% of chromosomal metaphases had 148 chromosomes with the karyotype formula of 38 m +60 sm +28 st +22 t (Figure 2E, J).

### DNA Content

The distribution of DNA content of all samples is shown in Table 2 and Figures 3A–E. The mean DNA content of 2nRT hybrids was equal (*P*>0.05) to the sum of half of RCC and TC, demonstrating that 2nRT hybrids had one set of chromosomes from both RCC and TC. The mean DNA content of 3nRT hybrids was equal (*P*>0.05) to the sum of RCC and half of TC, suggesting that 3nRT hybrids had two sets of chromosomes from RCC and one set of chromosomes from TC. The mean DNA content of 4nRT hybrids was equal (*P*>0.05) to that of RCC and TC, showing that 4nRT hybrids had two sets of chromosomes from both RCC and TC.

### Polymorphism of PCR Band Patterns

PCR amplification with 5S primers P_1_ and P_2_R for RCC, TC and their hybrid offspring produced distinctive band patterns. There were three bands (approximately 200, 350 and 500 bp) in RCC, two bands (approximately 200 and 300 bp) in TC, three bands (approximately 200, 350 and 500 bp) in 2nRT hybrids, three bands (approximately 200, 350 and 500 bp) in 3nRT hybrids, and four bands (approximately 200, 350, 400 and 500 bp) in 4nRT hybrids (Figure 4).

A total of 250 clones were sequenced to examine the different patterns of 5S rDNA, including 30 clones from RCC, 20 clones from TC, 60 clones from 2nRT hybrids, 60 clones from 3nRT hybrids and 80 clones from 4nRT hybrids (Table 3). Three fragments were indentified in the PCR product of RCC (203, 340 and 477 bp), two fragments occurred in TC (188 and 286 bp), four in 2nRT hybrids (179, 203, 340 and 495 bp), three in 3nRT hybrids (188, 340 and 495 bp), and four in 4nRT hybrids (203, 340, 406 and 495 bp) (Table 3). Sequence analysis showed that 2nRT hybrids presented two similarly sized 5S PCR products of 179 and 203 bp that were not distinguishable on the agarose gel, where they were seen as a single band of about 200 bp.

### Nucleotide Sequence Analysis of 5S rDNA

Using BLASTn, all fragments of RCC, TC and their filial generation were confirmed to be 5S rDNA repeat units, each comprising a 3' end of the 5S rRNA region (positions 1–21), a whole NTS region, and a large 5' flanking of the coding region of the adjacent unit (positions 22–120) (Figure 5A). In RCC, three fragments of 5S rDNA (designated class I: 203 bp; class II: 340 bp; and class III: 477 bp) were characterized by different NTS types (designated NTS–I, II, and III for the 83, 220, and 357 bp monomers, respectively). In TC, there were two fragments of 5S rDNA classes with the same coding region (designated class IV: 188 bp, and class V: 286 bp) but distinct NTS types (designated NTS–IV and NTS–V, for the 68 and 166 bp monomers, respectively) (Figure 5B). The sequence data demonstrated that RCC and TC showed high conservation in 5S coding regions and large variation in NTS regions. The mean G + C content of the coding regions in RCC and TC were 55% and 55.8%, respectively, both GC-rich. Comparative analysis of homology of the 5S rDNA fragments between hybrid offspring and their parents showed that the diploid hybrids (2nRT) had four 5S rDNA classes, with three (class I, II and III) deriving from their female parent (RCC) and one (class IV) from their male parent (TC). The triploid hybrids (3nRT) inherited three 5S rDNA classes, with two (class II and class III) from RCC and one (class IV) from TC. The tetraploid hybrids (4nRT) inherited two 5S rDNA classes (class II and class III) from RCC and presented a new 5S rDNA sequence (designated class I–N: 203 bp) with a novel NTS sequence (designated NTS–I–N: 83 bp). The specific paternal 5S rDNA sequence class V was not found in the hybrid offspring. In addition, the 406 bp DNA fragment from 4nRT hybrids was a dimeric 5S rDNA tandem array consisting of two class I–N sequences (Figure 5C). All the sequences, except the three that were shorter than 200 bp, were deposited in GenBank with the accession numbers JQ317900 to JQ317911.

All the internal control regions (including A box, internal element and C box) were identified in the coding regions of parents and their hybrid offspring. Although 5S rRNA genes were highly conserved in RCC and TC, several species-specific nucleotide variations were observed in the A box, positioned at 60, 61 and 63. Furthermore, among the hybrid progeny, several nucleotide variations were found not only in the ICRs but also at other four positions (Figure 6).

There were three NTS types in RCC (designated NTS–I, 83 bp; NTS–II, 220 bp; and NTS–III, 357 bp) and two NTS types in TC (designated NTS–IV, 68 bp, and NTS–V, 166 bp). Among the offspring, 2nRT hybrids had four types of NTS (NTS–I, NTS–II, NTS–III and NTS–IV), 3nRT hybrids possessed three types (NTS–II, NTS–III and NTS–IV), and 4nRT hybrids yet harbored three (NTS–I–N, NTS–II and NTS–III). The TATA box control element, upstream from the next array, was examined within all the NTS sequences of RCC, TC and their hybrid offspring, at positions −26 to −29 where it has been modified to TAAA (Figure 7). The thymidine residues necessary for transcription termination were also detected (Figures 6 and 7).

### Chromosomal Location of 5S rDNA

Through *in situ* hybridization, the 5S rDNA fluorescent probes prepared from the cloned 5S rDNA repeated units of TC (class IV and class V) were hybridized to the mitotic metaphase chromosomes of RCC, TC and their hybrid offspring. As shown in Figure 8, the probe from class IV of TC hybridized with the metaphase chromosomes of TC, 2nRT and 3nRT hybrids, but not with RCC and 4nRT hybrids; the probe from class V of TC hybridized only with the metaphase chromosomes of TC. These results and the data in Table 3 indicated the different heredity characteristic of paternal-specific 5S rDNA.

## Discussion

### Formation of Diploid, Triploid and Tetraploid Hybrids

The different ploidy levels of hybrid fish were determined by counting the chromosomes and examining DNA content using flow cytometry. The former is a direct and accurate method for determining ploidy of samples, and the latter is a rapid, simple and equally accurate method.

Both chromosome number and karyotype formula of RCC were similar to the results of previous studies [11]–[12]. 2nRT, 3nRT and 4nRT hybrids possessed 74, 124 and 148 chromosomes respectively (Figure 2), in contrast to RCC (100) and TC (48). Furthermore, it is apparent that a pair of the largest submetacentric chromosomes in diploid TC can be used as marker chromosomes for identifying TC from RCC. Possessing 74 chromosomes and a submetacentric largest chromosome, 2nRT hybrids were proved to have obtained one set of chromosomes from RCC and one set of chromosomes from TC (Figure 2C, H). With 124 chromosomes and a submetacentric largest chromosome, 3nRT hybrids apparently contain two sets of chromosomes from RCC and one set of chromosomes from TC (Figure 2D, I). 4nRT hybrids had 148 chromosomes and one pair of submetacentric chromosomes and are suggested to harbor two sets of chromosomes from RCC and two sets of chromosomes from TC (Figure 2E, J). In addition, there was a significant relationship between the sum of the mean DNA content of the hybrid offspring and that of their parents (Figure 3, Table 2). These results indicate the hybridization origin of the progeny of RCC♀×TC♂, rather than gynogenesis or androgenesis.

Polyploidization is an important and frequent event in lower vertebrate evolution, especially that of fish [46]–[47]. In our previous studies, we successfully obtained two kinds of bisexual fertile tetraploid hybrids (4 nAT and 4 nRB) [11]–[12]. In the case of 4 nAT, there were only diploid hybrids with 100 chromosomes in the F_1_–F_2_ generation, while the tetraploid hybrids with 200 chromosomes were found in F_3_ and the later generations, being formed via the fusion of unreduced gametes. For 4 nRB, only 3 nRB and 4 nRB hybrids were formed, and no diploid hybrids were found to survive past the first generation (although diploid embryos were observed). 3 nRB hybrids resulted from the retention of the second polar body, whereas 4 nRB hybrids resulted from somatic doubling. Recently, the coexistence of diploid, triploid and tetraploid crucian carp (*Carassius auratus*) in natural waters has been reported [48]. In the present study, viable 2nRT, 3nRT and 4nRT hybrids were created for the first time by crossing different parents (RCC and TC) belonging to distinct subfamilies with different chromosome numbers. We hypothesize that the different ploidy numbers of the hybrid offspring arose by non-disjunctive or incomplete cell division during the first doubling.

### Molecular Organization of the 5S rDNA

Numerous studies have reported the retention of more than one 5S rDNA repeat class and the elimination of parental-specific 5S rDNA following polyploidization in plants and fishes [40], [49]–[55]. As previously reported by Qin et al. (2010), RCC was found to have three types of 5S rDNA sequences owing to a probably ancient polyploidization. 3 nRB and 4 nRB hybrids possessed four and three types of 5S rDNA sequences, respectively, following allopolyploidization, and a paternal-specific loss of 5S rDNA was also observed in 4 nRB hybrids. A similar result was found in the present study, with three types of 5S rDNA sequences observed in RCC having high similarity (100%, 99.7% and 98.7%, respectively) to sequences in GenBank (GQ485555, GQ485556 and GQ485557) which were uploaded by Qin et al. (2009). Moreover, only two types of 5S rDNA sequences were detected in TC, favoring the hypothesis that the presence of two classes of 5S rDNA is a general trend for the 5S rRNA gene organization in the fish genome [38]. However, 2nRT, 3nRT and 4nRT hybrids had four, three and three types of 5S rDNA sequences, respectively, partially inheriting 5S rDNA classes from their parents. FISH results (Figure 8) also confirmed the loss of paternal-specific 5S rDNA classes. Moreover, the internal control regions (A box, IE and C box), TATA-like control element and thymidine residues which are necessary for the correct gene expression, were observed in all the sequences of the parents and their hybrid offspring, indicating that all sequences analyzed here were functional genes.

Hybridization can result in genomic changes including alterations of gene expression, chromosomal structure, and genome size [56]. In the present work, we detected several nucleotide mutations in the coding regions of the hybrid progeny, even in the ICRs (Figure 6). We also observed nucleotide polymorphisms including base constitution and insertion-deletion in the NTS sequences of the hybrid offspring (Figure 7).

It has been reported that the NTS sequence could be employed as a molecular marker for species identification and phylogenetic studies [17], [35], [40], [57]–[60]. Here, three NTS types (NTS–I, NTS–II and NTS–III) with different length and base constitutions were detected in RCC. TC had two types of distinct NTS (NTS–IV and NTS–V) (Figure 7). Four types of NTS (NTS–I, NTS–II, NTS–III and NTS–IV) were detected in 2nRT hybrids, with types III and IV resulting from an insertion in the NTS-III sequence and a deletion in the NTS-IV sequence, respectively. The 3nRT hybrids had three NTS types (NTS–II, NTS–III and NTS–IV), and the same insertion was also detected in NTS–III. There were three types of NTS (NTS–I–N, NTS–II and NTS–III) in 4nRT hybrids, with NTS–I–N having several base mutations. These variations in NTS sequences indicate that 5S rDNA can serve as a suitable genetic marker for evolutionary studies and for the genetic identification of hybrid fish and their parent species.

In previous studies, a minimum length (59 bp) of NTS sequence was found to maintain the array and the expression/regulation dynamic of 5S rRNA genes in the fish genome [38], [61]–[62]. 2nRT hybrids inherited nearly all the 5S rDNA classes from their parents (with the exception of the class V 5S rDNA of TC). Furthermore, almost all the 5S rDNA classes preserved the structural organization characteristic of their parents (with the exception of a deletion of a T at position −59 and a sequence of GAATACCT at positions −30 to −37 in the NTS–IV sequences of TC) (Figure 7). It’s the first time to describe this finding in F_1_ hybrids of fish and provides further evidence that a 59 bp length of NTS sequence is the minimum necessary to guarantee the array and the dynamics of the 5S rRNA genes.

In this study, 4nRT hybrids lost all the paternal-specific 5S rDNA classes (IV and V) and presented a newly formed allopolyploid-special 5S rDNA sequence (class I–N). This novel 5S rDNA sequence showed a low similarity to that of their parents but a high homology to that of common carp (*Cyprinus carpio*, CC, Accession number: AB015590), even though this species belongs to a different genus (Figure 7, Table 4). It’s the first report of this phenomenon in the cross of RCC♀×TC♂. Similar findings have previously been reported [12], [40], [63]. For example, the barbels and the Sox gene, which were special traits for CC, presented in 4 nRB hybrids but were absent in 3 nRB hybrids and their parents (RCC and BSB). Importantly, there was also an allopolyploid-special 5S rDNA sequence (Accession number: GU329957) of 4 nRB hybrids with low similarity to that of their parents but high homology to that of CC (Accession number: AB015590). The formation of CC-specific DNA sequences and genes in allopolyploid hybrids imply that hybridization and allopolyploidization could result in rapid genomic changes in the hybrid offspring of RCC♀×TC♂. In addition, it has been reported that a drastic elimination and recombination of the parental-specific rDNA repeat units has some relationship to the fertility of newly formed hybrids or allopolyploids [55], [64]–[65]. Thus, to improve fertility, rapidly genetic recombination was required in 4nRT hybrids, which led to the absence of parental-specific 5S rDNA and the presence of a novel 5S rDNA. Consequently, we can assume that 4nRT hybrids were fertile, although confirmation of fertility or sterility requires further study. However, there was no data available to allow interpretation of the possible evolutionary relationship between CC and the allopolyploid hybrids.

In conclusion, this is the first report on the formation of these viable diploid, triploid and tetraploid hybrids by crossing different parents with a different chromosome number in vertebrates. Results observed here confirmed the influence of hybridization and allopolyploidization on the 5S rDNA organization, and provided new information on the genetic variation of the 5S rDNA multigene family in vertebrates. We will continue the comparative study of genomic and phenotypic patterns in the hybrid offspring in our future studies.
